# Supplementary material for: Physico-Mechanical Optimization and Antimicrobial Properties of the Bionanocomposite Films Containing Gallic Acid and Zinc Oxide Nanoparticles
Source: Nanomaterials (Basel). 2023 May 31;13(11):1769. doi: 10.3390/nano13111769 (PMC10254433; doi:10.3390/nano13111769)
Supplement: Supplementary file 1 [file nanomaterials-13-01769-s001.zip › nanomaterials-2338857-supplementary.pdf]

Supporting Information

Physico-Mechanical Optimization and Antimicrobial Properties of the Bionanocomposite Films Containing Gallic Acid and Zinc Oxide Nanoparticles

Azin Karami [1], Babak Ghanbarzadeh [1,2,*], Leila Abolghasemi Fakhri [1], Pasquale M. Falcone [3,*] and Mohammadyar Hosseini [4]

[1] Department of Food Science and Technology, Faculty of Agriculture, University of Tabriz, P.O. Box 51666-16471, Tabriz, Iran

[2] Department of Food Engineering, Faculty of Engineering, Near East University, Nicosia P.O. Box 99138, Cyprus, Mersin 10, Turkey

[3] Department of Agricultural, Food and Environmental Sciences, University Polytechnical of Marche, Brecce Bianche 10, 60131 Ancona, Italy

[4] Department of Food Science and Hygiene, Faculty of Para-Veterinary, Ilam University, Ilam, Iran

* Correspondence: authors: ghanbarzadeh@tabrizu.ac.ir (B.G.); pm.falcone@univpm.it (P.M.F.)

Table S1. The adequacy of models tested (lack of fit tests and sequential model sum of squares)

Sequential model sum of squares					Lack of fit test			
**Sourse**	**df**	**F-Value**	**p-value Prob>F**	**Remark**	**df**	**F-Value**	**p-value Prob>F**	**Remark**
YI								
Linear	2	14.51	0.0011**		6	21.01	0.0054**	
2F1	1	0.88	0.3737		5	22.91	0.0048**	
Quadratic	2	60.35	<0.0001**	Suggested	3	0.83	0.5415	Suggested
Residual	5				-			
Total	13				-			
L								
Linear	2	15.08	0.0010**		6	74.19	0.0005**	
2F1	1	0.65	0.4412		5	82.98	0.0004**	
Quadratic	2	76.98	<0.0001**	Suggested	3	4.74	0.0835	Suggested
Residual	5				-			
Total	13				-			
ΔE								
Linear	2	15.31	0.0009**		6	23.37	0.0044**	
2F1	1	0.47	0.5098		5	26.61	0.0036**	
Quadratic	2	72.11	<0.0001**	Suggested	3	0.78	0.5630	Suggested
Residual	5				-			
Total	13				-			
UTS								
Linear	2	48.08	<0.0001**	Suggested	6	5.39	0.0625	Suggested
2F1	1	0.67	0.4333		5	5.96	0.0541	
Quadratic	2	0.93	0.4395		3	7.58	0.0398	
Residual	5				-			
Total	13				-			
S_{max}								
Linear	2	21.07	0.0003**		6	7.57	0.0352	
2F1	1	17.21	0.0025**		5	2.60	0.1882	
Quadratic	2	4.76	0.0495*	Suggested	3	1.06	0.4574	Suggested
Residual	5				-			
Total	13				-			
YM								
Linear	2	21.89	0.0002**		6	1.31	0.4159	
2F1	1	4.91	0.0538	Suggested	5	0.73	0.6370	Suggested
Quadratic	2	2.11	0.2125		3	0.26	0.8536	
Residual	5				-			
Total	13				-			
WVP								
Linear	2	16.37	0.0007**		6	7.04	0.0399	
2F1	1	5.16	0.0493		5	5.08	0.0703	
Quadratic	2	12.49	0.0049**	Suggested	3	0.81	0.5504	Suggested
Residual	5				-			
Total	13				-			

*, **:Significant at p<5% and p<1%, respectively

Table S2. The adequacy of models tested (Model summary statistics)

Sourse	Std.Dev.	R-Squared	Adj R-Squared	Pred R-Squared	Press	Remark
YI						
Linear	8.93	0.7437	0.6924	0.5828	1299.41	
2F1	8.98	0.7664	0.6886	0.4720	1641.91	
Quadratic	2.38	0.9872	0.9781	0.9527	147.14	Suggested
L						
Linear	3	0.7509	0.7011	0.5929	146.82	
2F1	3.05	0.7677	0.6903	0.4167	210.38	
Quadratic	0.72	0.9899	0.9827	0.9405	21.47	Suggested
ΔE						
Linear	3.14	0.7538	0.7046	0.6046	158.04	
2F1	3.22	0.7660	0.6881	0.4589	216.26	
Quadratic	0.79	0.9892	0.9814	0.9609	15.64	Suggested
UTS						
Linear	0.39	0.9058	0.8870	0.8081	3.13	Suggested
2F1	0.40	0.9124	0.8831	0.7436	4.18	
Quadratic	0.40	0.9307	0.8812	0.5648	7.09	
S_{max}						
Linear	3.22	0.8082	0.7699	0.6136	208.72	
2F1	1.99	0.9341	0.9122	0.8713	69.49	
Quadratic	1.47	0.9721	0.9522	0.8877	60.68	Suggested
YM						
Linear	14.51	0.8141	0.7769	0.6833	3587.87	
2F1	12.30	0.8797	0.8397	0.8069	2187.16	Suggested
Quadratic	11.01	0.9250	0.8715	0.8157	2087.60	
WVP						
Linear	1.929E-011	0.7660	0.7192		+	
2F1	1.621EE-011	0.8513	0.8017		+	
Quadratic	8.601E-012	0.9674	0.9442	0.8808	1.896E-021	Suggested

Table S3. Responses optimization

Variables and response	Aim	Low limit	High limit	degree of importance
Zno (%)	In range	0	2	3
GA (%)	In range	5.01	15.04	3
WVP (g/msPa)	Min	1.25×10^{-11}	8.67×10^{-11}	4
L	Max	40.25	65.25	3
ΔE	Min	17.79	36.237	3
YI	Min	39.93	91.3613	3
UTS (Mpa)	Max	2.93	7.52	5
S_{max} (%)	Max	17.11	41.53	5
YM (Mpa)	Max	20.7	120.4	4
